# Machine learning to predict distant metastasis and prognostic analysis of moderately differentiated gastric adenocarcinoma patients: a novel focus on lymph node indicators

**DOI:** 10.3389/fimmu.2024.1398685

**Published:** 2024-09-19

**Authors:** Kangping Yang, Jiaqiang Wu, Tian Xu, Yuepeng Zhou, Wenchun Liu, Liang Yang

**Affiliations:** ^1^ Department of Gastroenterological Surgery, The Second Affiliated Hospital, Jiangxi Medical College, Nanchang University, Nanchang, Jiangxi, China; ^2^ Department of General Surgery, First Medical Center of the Chinese People's Liberation Army General Hospital, Beijing, China; ^3^ Department of Gastroenterological Surgery, Jiangxi Hospital of Integrated Traditional Chinese and Western Medicine, Nanchang, Jiangxi, China; ^4^ The Second Department of Internal Medicine, Anfu People’s Hospital, Anfu, Jiangxi, China

**Keywords:** moderately differentiated gastric adenocarcinoma, prognosis, nomogram, lymph node indicators, distant metastasis, machine learning

## Abstract

**Background:**

Moderately differentiated gastric adenocarcinoma (MDGA) has a high risk of metastasis and individual variation, which strongly affects patient prognosis. Using large-scale datasets and machine learning algorithms for prediction can improve individualized treatment. The specific efficacy of several lymph node indicators in predicting distant metastasis (DM) and patient prognosis in MDGA remains obscure.

**Methods:**

We collected data from MDGA patients from the SEER database from 2010 to 2019. Additionally, we collected data from MDGA patients in China. We used nine machine learning algorithms to predict DM. Subsequently, we used Cox regression analysis to determine the risk factors affecting overall survival (OS) and cancer-specific survival (CSS) in DM patients and constructed nomograms. Furthermore, we used logistic regression and Cox regression analyses to assess the specific impact of six lymph node indicators on DM incidence and patient prognosis.

**Results:**

We collected data from 5,377 MDGA patients from the SEER database and 109 MDGC patients from hospitals. T stage, N stage, tumor size, primary site, number of positive lymph nodes, and chemotherapy were identified as independent risk factors for DM. The random forest prediction model had the best overall predictive performance (AUC = 0.919). T stage, primary site, chemotherapy, and the number of regional lymph nodes were identified as prognostic factors for OS. Moreover, T stage, number of regional lymph nodes, primary site, and chemotherapy were also influential factors for CSS. The nomograms showed good predictive value and stability in predicting the 1-, 3-, and 5-year OS and CSS in DM patients. Additionally, the log odds of a metastatic lymph node and the number of negative lymph nodes may be risk factors for DM, while the regional lymph node ratio and the number of regional lymph nodes are prognostic factors for OS.

**Conclusion:**

The random forest prediction model accurately identified high-risk populations, and we established OS and CSS survival prediction models for MDGA patients with DM. Our hospital samples demonstrated different characteristics of lymph node indicators in terms of distant metastasis and prognosis.

## 1 Introduction

Gastric cancer, a very prevalent gastrointestinal tumor, is the fifth most prevalent tumor worldwide (1). In 2020, there were more than one million additional cases of gastric cancer (2). The histologic type of gastric cancer is predominantly adenocarcinoma, and the pathologic grade includes highly, moderately, and poorly differentiated and undifferentiated (3, 4). Although progressive gastric cancer is predominantly poorly differentiated, some moderately differentiated gastric adenocarcinomas (MDGAs) still have a high risk of metastasis and individual differences, which have been reported in animal models and clinical studies (5–7). There is no doubt that the occurrence of distant metastasis (DM) directly affects patient prognosis (8). According to the latest eighth revision of the UICC/AJCC TNM classification for gastric cancer, once DM occurs, the disease has already entered stage IV, at which time the patient’s survival chances are extremely poor (9). A retrospective study showed that the median overall survival (OS) time for patients with liver metastases from gastric cancer was 7 months and that for patients with lung and brain metastases (10) was only 5 months. Timely and accurate determination of the distant metastasis status of gastric cancer patients has important positive implications for avoiding missing opportunities for early and effective interventions and improving patient survival.

Currently, tests to clarify the occurrence of DM mainly rely on multidetector computed tomography (CT), positron emission tomography-CT (PET/CT), and other imaging methods (11, 12). However, all of these methods have the problem of insufficient sensitivity in practical applications (13). For example, in PET/CT, some poorly differentiated carcinomas, mucinous carcinomas, and indolent cell carcinomas usually have low ^18^F-FDG uptake, which often results in false-negative results and delayed therapy (14). Therefore, there is an urgent need for an accurate, convenient, yet affordable method for DM diagnosis and prediction. The use of emerging machine learning (ML) algorithms and large-scale datasets to construct predictive models is currently a popular solution (15–17). ML algorithms are able to accurately process raw data originating from databases, analyze the relationships between important data, and ultimately build and filter the best predictive models (18–21). This prediction model, which integrates clinical manifestations and imaging data to form a comprehensive assessment tool, can be used to diagnose the presence or absence of DM early and accurately and can better guide subsequent clinical diagnosis and treatment.

For patients with already occurring DM, the median OS after performing conventional chemotherapy is approximately 12 months (22). With regard to cancer-specific survival (CSS), the 1- and 3-year CSS rates for the younger group (≤60 years of age) were 29.0% and 6.2%, respectively, compared with 22.8% and 4.8% for the older group (>60 years of age), respectively (23). These findings suggest that there are many factors that can influence DM patient prognosis, and clarifying the effects of these factors and applying them in a targeted manner are important ways to improve patient prognosis. Many studies have demonstrated that factors such as age, tumor size, sex, degree of differentiation, and primary site are directly associated with DM patient prognosis (24–26). Moreover, recent studies have demonstrated a strong association between various lymph node indicators and DM and the prognosis of moderately differentiated gastric adenocarcinoma patients. For example, lymph node-specific indicators include the number of positive lymph nodes (PLNs), the lymph node ratio (LNR), and the log odds of metastatic LNs (LODDS) (27–29). However, the specific efficacy of these lymph node indicators in predicting DM and patient prognosis is unclear (30–33). This study explored these prognostic factors in DM patients in the MDGA to provide strong theoretical support for individualized treatment in this population. Afterward, the above factors were combined to construct OS and CSS prognostic nomograms at 1, 3, and 5 years for DM patients with MDGA, which is a simplified visualization model for statistical prediction in combination with independent factors.

Our goal was to formulate models for predicting DM in MDGA patients and to ensure the stability and accuracy of these models through both database validation and external validation. A prognostic analysis of DM patients was then performed to plot OS and CSS prognostic nomograms for MDGA patients. Importantly, we focused on exploring the relationships between various lymph node indicators whose efficacy is still unclear and between DM and prognosis to further promote the application of lymph node indicators in the clinical practice of stomach cancer diagnosis.

## 2 Materials and methods

### 2.1 Sources of data and sample selection

**Figure 1:**
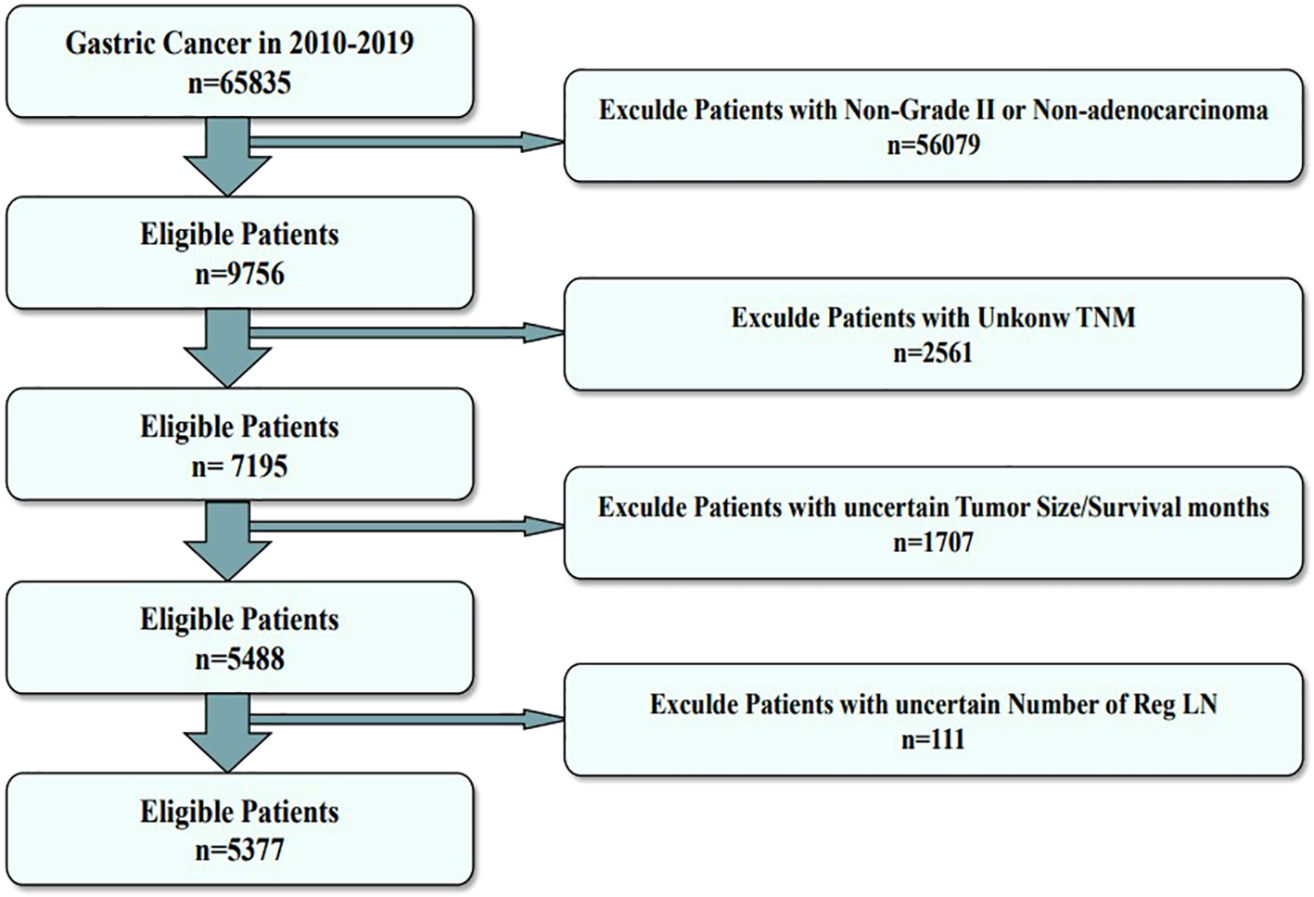
Flowchart of the data screening process. The figure shows the process of filtering eligible patient data from the SEER database.

The primary training dataset was obtained by collecting all 2010–2019 gastric cancer patient data from the Surveillance, Epidemiology, and End Results (SEER) database. The SEER database is the most detailed publicly available cancer database. Moreover, we collected the clinical data of MDGA patients treated at the Second Affiliated Hospital of Nanchang University between 2008 and 2010 as an external validation dataset. The inclusion criteria were as follows: 1) had a diagnosis of MDGA, 2) did not receive preoperative radiotherapy or immunotherapy, and 3) had comprehensive and searchable prognostic data. The exclusion criteria were as follows: 1) patients whose primary tumor was not gastric cancer, 2) patients whose tumor and lymph node status were not clear, and 3) patients whose other basic information was incomplete. The specific data selection steps are illustrated below in **Figure 1**.

### 2.2 Variable selection

Variables in the present study included age, TNM stage, primary site, tumor size, sex (male or female), and two therapeutic variables (chemotherapy and radiation) obtained from the diagnostic information, as well as several lymph node indicators. Multiple lymph node indicators included the number of Reg LNs, number of all LNs, number of Reg LNs, number of Neg LNs, gross LN metastasis, LN positivity rate, log odds of metastatic LNs, and lymph node ratio (number of metastatic LNs to total number of LNs examined).

OS and CSS are the main outcomes for predicting the prognosis of patients with DM. In OS, deaths due to any cause will be counted, while in CSS analysis, only deaths due to MDGA will be considered events, and deaths due to other factors as well as survival will be excluded.

### 2.3 Statistical methods

**Figure 2:**
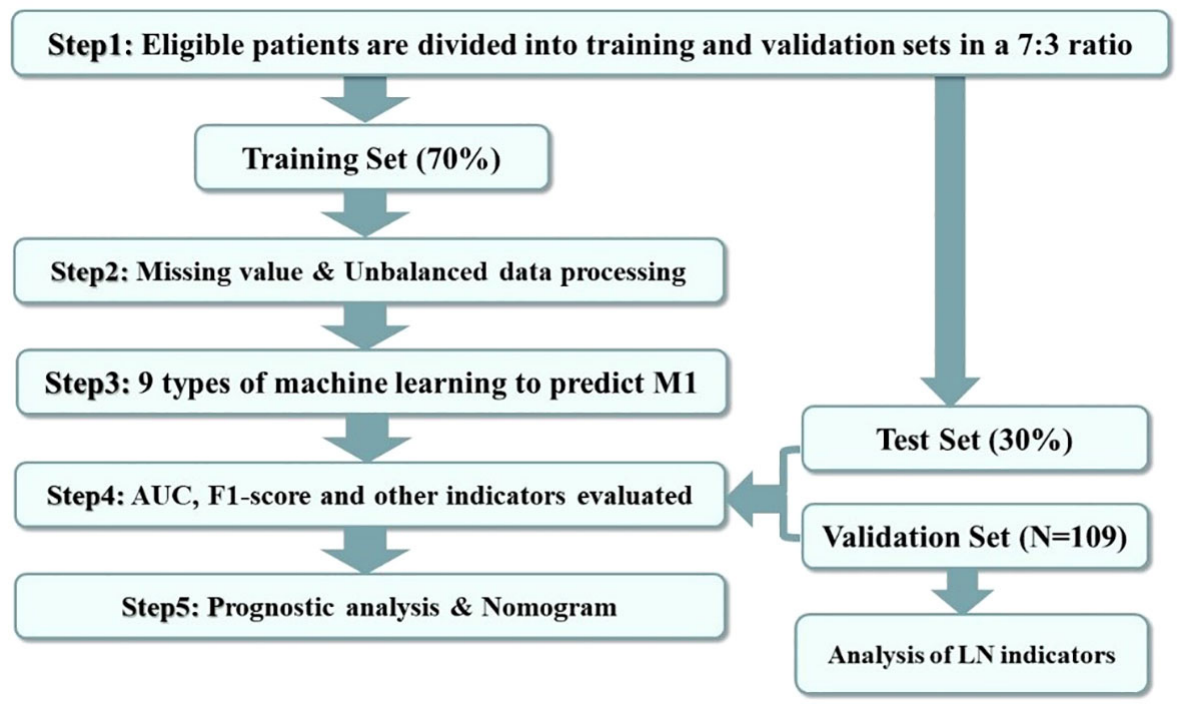
Data analysis guide. The figure shows the procedure of this study for processing and analyzing the screened data.

The research procedure is illustrated in **Figure 2**. Heatmapping was first developed to correlate the proposed study variables. We use regression analysis and machine learning for dual validation of risk factors; regression analysis is performed using the full SEER data, and machine learning uses the training set, the test set, and the external validation set to construct predictive models. Independent risk factors influencing DM in moderately differentiated gastric adenocarcinoma patients were screened by logistic regression analysis. The outcomes are expressed as hazard ratios (HRs) and 95% confidence intervals (CIs). The patient data screened from the SEER dataset were randomized 7:3 into a training set and a test set. Then, the training set will be utilized to build the predictive model. The constructed predictive models are then tested and evaluated using the test set data. We constructed nine ML algorithms in the training set, including RF (random forest), LR (logistic regression), LASSO (least absolute shrinkage and selection operator), SVM (support vector machine), KNN (K-nearest neighbor), NBC (naive Bayes classifier), and ANN (artificial neural network). The receiver operating characteristic (ROC) curve, the area under the ROC curve (AUC), sensitivity, specificity, F1 score, and accuracy were used to compare the performance of the models. Additionally, the predictive models were evaluated and validated using test set data. Self-collected hospital patient data were used as an external validation set to validate the best predictive model that assessed the generalization ability of the model.

We used several R packages in R for data analysis and visualization. The createDataPartition function of the caret package was used for grouping the training and validation sets. The imp function of randomForest package was used to construct the importance scores of RF. The coords function of the pROC package was used to construct the confusion matrix. The randomForest package, the MASS package, the rms package, the glmnet package, the e1071 package, the xgboost package, the adabag package, and the neuralnet package were all used for machine learning model construction. The MASS package, rms package, glmnet package, e1071 package, xgboost package, adabag package, and neuralnet package were used for the construction of machine learning models. The ggplot package and pROC package were used for the visualization of ROC curves and importance scores.

For survival prognostic analyses, single-variable Cox regression analysis was first adopted to screen the relevant variables that could influence the prognosis (*P* < 0.05), and then multifactorial analyses were carried out on the screened variables. Moreover, we used the Kaplan−Meier curves to assess the differences in survival prognosis among patients stratified by different variables and compared the results by means of the log-rank test. The independent risk factors identified through Cox regression analysis were used to construct the nomogram. Moreover, using multifactor Cox regression analysis, the regression coefficients *β* (coe *β*) for each variable were normalized and are displayed as risk scores on the nomograms. The accuracy and discriminatory power of the generated nomograms were assessed with the AUC, calibration curves, and consistency index (C-index). In addition, we evaluated the clinical value of the nomograms by using decision curve analysis (DCA). This is a commonly used measure to assess model validity by quantitatively estimating the net effectiveness under the exposure threshold.

Finally, the impact of multiple nuanced tumor-associated LN indicators on the development of DM in MDGA patients was investigated by logistic regression analysis of patient data collected at our institution, as well as factors affecting patient prognosis. For descriptive statistics, categorical variables were compared using the chi-square test or Fisher’s exact probability method. *P <*0.05 indicated statistical significance.

### 2.4 Ethics approval

The use of patient data in this research has been authorized. The approval document from the Ethics Committee is shown in the attachment. Patients from the SEER database provided consent for open research in any scientific study worldwide.

## 3 Results

### 3.1 Basic features and patient subgroups

Altogether, 5,377 patients from the database were included in this study; 749 (13.93%) had DM, and 4,628 (86.07%) had no DM. The local patient dataset, which served as an external validation set, included a total of 109 patients, of whom 15 (13.76%) had DM and 94 (86.24%) had no DM. The patient data screened from the SEER dataset were randomized 7:3 into training and testing sets. The results of the analyses, as shown in **Table 1**, show the comprehensive demographic and clinical characteristics of the three groups of MDGA patients. Additionally, there were no statistically significant differences (*P* > 0.05) in any of the clinical characteristics analyzed, such as tumor size, primary site, TNM stage, or number of Reg LNs, between the patients in the training and testing sets.

**Table 1:** Comparison of the general features of the training and test sets.

Variable	Training set (%) *N* = 3,764	Test set (%) *N* = 1,613	*P*	Validation set (%) *N* = 109
Age (years)			0.800	
<40	42 (1.1%)	23 (1.4%)		4
40–60	675 (17.9%)	283 (17.5%)		35
60–80	2,215 (58.8%)	948 (58.8%)		67
>80	832 (22.1%)	359 (22.3%)		3
Sex			0.443	
Male	2,673 (71.0%)	1,128 (69.9%)		83
Female	1,087 (29.0%)	489 (30.1%)		26
T stage			0.612	
1	1,364 (36.2%)	573 (35.5%)		23
2	561 (14.9%)	230 (14.3%)		17
3	1,334 (35.4%)	573 (35.5%)		55
4	505 (13.4%)	237 (14.7%)		14
N stage			0.997	
0	2,012 (53.5%)	865 (53.6%)		45
1	976 (25.9%)	417 (25.9%)		40
2	471 (12.5%)	199 (12.3%)		12
3	305 (8.1%)	132 (8.2%)		12
M stage			0.919	
0	3,238 (86.0%)	1,390 (86.2%)		94
1	526 (14.0%)	223 (13.8%)		15
Primary site			0.419	
Body	303 (8.0%)	143 (8.9%)		7
Cardia	1,509 (40.1%)	650 (40.3%)		40
Fundus	134 (3.6%)	59 (3.7%)		2
Gastric antrum	817 (21.7%)	378 (23.4%)		30
Greater curvature	141 (3.7%)	50 (3.1%)		2
Lesser curvature	342 (9.1%)	131 (8.1%)		8
Overlapping lesion	182 (4.8%)	77 (4.8%)		12
Pylorus	136 (3.6%)	43 (2.7%)		4
Stomach	200 (5.3%)	82 (5.1%)		4
Tumor size (cm)			0.280	
<2	971 (25.8%)	457 (28.3%)		24
2 to 5	1792 (47.6%)	744 (46.1%)		51
5 to 8	766 (20.4%)	312 (19.3%)		23
>8	235 (6.2%)	100 (6.2%)		11
Number of Reg LN			0.997	
None	1,204 (32.0%)	515 (31.9%)		47
1 to 3	167 (4.4%)	71 (4.4%)		22
4 or more	2,393 (63.6%)	1,027 (63.7%)		40

### 3.2 Comparison and analysis of model variables

**Figure 3:**
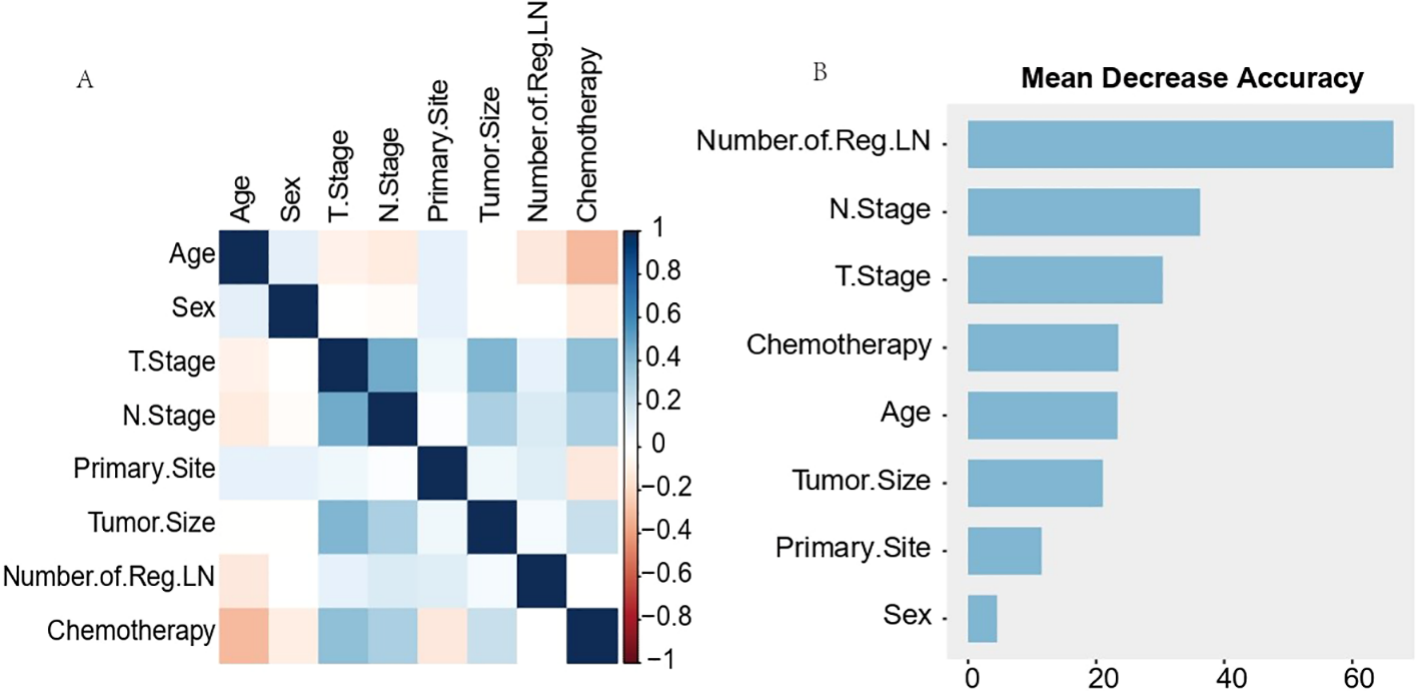
Results of Pearson correlation analysis for each variable **(A)** and ranking of importance of predictive model characteristics **(B)**. The results of Pearson correlation analysis for each variable **(A)** showed that all variables existed independently of each other. The predictive model characteristics **(B)** were the number of Reg LNs, N stage, T stage, chemotherapy, age, tumor size, and primary site, in order of importance.

Pearson correlation analysis was used to examine the relationship between each variable (**Figure 3A**). Moreover, with the comprehensive consideration of the type of data, distribution characteristics, and other factors, all the variables are independent and well-distributed and can be included in the following statistical analysis. By multifactorial logistic regression analysis, this study revealed that six variables were statistically significant in predicting the occurrence of DM in patients with MDGA (**Table 2**). These included the T and N stages, but the M stage seemed to be not significantly different. Other variables included primary site, tumor size, number of Reg LNs, and chemotherapy. In addition, the importance scores from the random forest model indicated variable significance (as displayed in **Figure 3B**). The number of Reg LNs, N stage, T stage, chemotherapy, age, tumor size, and primary site were positively related to the occurrence of DM in MDGA patients. Specifically, the outcome was the same as the findings of the former correlation analyses, except for age. With distant metastasis as the outcome variable, we conducted single- and multiple-factor logistic regression analyses on eight factors: primary site, tumor size, age, sex, T stage, N stage, number of positive LNs, and chemotherapy. Multiple factor regression was performed, and step-forward analysis revealed that the *P*-values for T stage, N stage, primary site, number of positive LNs, tumor size, and chemotherapy were less than 0.05 and were considered statistically significant independent risk factors. The results of forward regression analysis indicated the meaningful impact of six variables on distant metastasis: sex, T stage, N stage, primary site, tumor size, and number of positive LNs (the detailed results are presented in the **Supplementary Material**).

**Table 2:** Multifactorial analysis of moderately differentiated distant metastatic gastric adenocarcinoma.

Variables	Beta	S.E	*Z*	OR (95% CI)	*P*	aBeta	aS.E	a*Z*	aOR (95% CI)	a*P*
Age										
<40				1.00 (reference)						
40–60	−0.26	0.37	−0.71	0.77 (0.37–1.59)	0.478					
60–80	−0.60	0.36	−1.64	0.55 (0.27–1.12)	0.100					
>80	−0.62	0.37	−1.67	0.54 (0.26–1.11)	0.094					
Sex										
Male				1.00 (reference)						
Female	−0.16	0.11	−1.53	0.85 (0.69–1.05)	0.125					
T stage										
1				1.00 (reference)					1.00 (reference)	
2	−0.55	0.19	−2.90	0.58 (0.40–0.84)	0.004	−0.69	0.21	−3.27	0.50 (0.33–0.76)	0.001
3	0.25	0.12	2.12	1.28 (1.02–1.61)	0.034	−0.19	0.15	−1.25	0.82 (0.61–1.12)	0.210
4	1.25	0.13	9.72	3.49 (2.71–4.49)	<0.001	0.68	0.17	3.94	1.97 (1.41–2.76)	<0.001
N stage										
0				1.00 (reference)					1.00 (reference)	
1	1.12	0.11	10.19	3.08 (2.48–3.82)	<0.001	1.00	0.14	7.16	2.73 (2.07–3.60)	<0.001
2	0.59	0.16	3.75	1.80 (1.32–2.44)	<0.001	0.89	0.20	4.52	2.45 (1.66–3.60)	<0.001
3	1.23	0.16	7.83	3.42 (2.52–4.66)	<0.001	1.84	0.21	8.76	6.29 (4.17–9.49)	<0.001
Primary site										
Cardia				1.00 (reference)					1.00 (reference)	
Gastric antrum	−0.16	0.13	−1.22	0.85 (0.66–1.10)	0.222	0.58	0.17	3.49	1.78 (1.29–2.47)	<0.001
Lesser curvature	−0.29	0.19	−1.57	0.75 (0.52–1.07)	0.116	0.21	0.22	0.98	1.24 (0.81–1.89)	0.326
Pylorus	−0.85	0.35	−2.41	0.43 (0.21–0.85)	0.016	−0.11	0.40	−0.29	0.89 (0.41–1.94)	0.772
Body	0.14	0.17	0.84	1.15 (0.83–1.60)	0.400	0.58	0.20	2.88	1.79 (1.20–2.66)	0.004
Greater curvature	0.08	0.24	0.31	1.08 (0.67–1.74)	0.754	0.36	0.29	1.22	1.43 (0.81–2.52)	0.222
Stomach	0.30	0.20	1.54	1.35 (0.92–1.99)	0.124	0.83	0.24	3.52	2.29 (1.44–3.63)	<0.001
Overlapping lesion	0.11	0.22	0.51	1.12 (0.73–1.70)	0.611	0.08	0.26	0.32	1.09 (0.65–1.81)	0.746
Fundus	0.13	0.23	0.56	1.14 (0.72–1.80)	0.579	0.13	0.28	0.45	1.13 (0.66–1.95)	0.649
Tumor size										
<2				1.00 (reference)					1.00 (reference)	
2 to 5	1.11	0.15	7.18	3.02 (2.23–4.09)	<0.001	0.85	0.17	4.93	2.35 (1.67–3.30)	<0.001
5 to 8	1.55	0.17	9.35	4.69 (3.39–6.49)	<0.001	1.24	0.19	6.38	3.44 (2.36–5.04)	<0.001
>8	1.75	0.20	8.68	5.75 (3.87–8.54)	<0.001	1.15	0.24	4.78	3.15 (1.97–5.04)	<0.001
Number of Reg LN										
None				1.00 (reference)					1.00 (reference)	
1 to 3	−1.18	0.24	−4.88	0.31 (0.19–0.49)	<0.001	−1.38	0.27	−5.22	0.25 (0.15–0.42)	<0.001
4 or more	−2.08	0.11	−18.95	0.12 (0.10–0.15)	<0.001	−2.75	0.14	−19.25	0.06 (0.05–0.08)	<0.001
Chemotherapy										
No/unknown				1.00 (reference)					1.00 (reference)	
Yes	0.66	0.10	6.90	1.94 (1.61–2.35)	<0.001	0.36	0.12	2.92	1.43 (1.13–1.82)	0.003

### 3.3 Establishment of predictive models for distant metastasis

This research used nine distinct machine learning models individually to construct a distant metastasis prediction model for MDGA patients. The built models were trained with data from the training set. The symptoms were finely tuned to stabilize the models and prevent them from overfitting.

**Figure 4:**
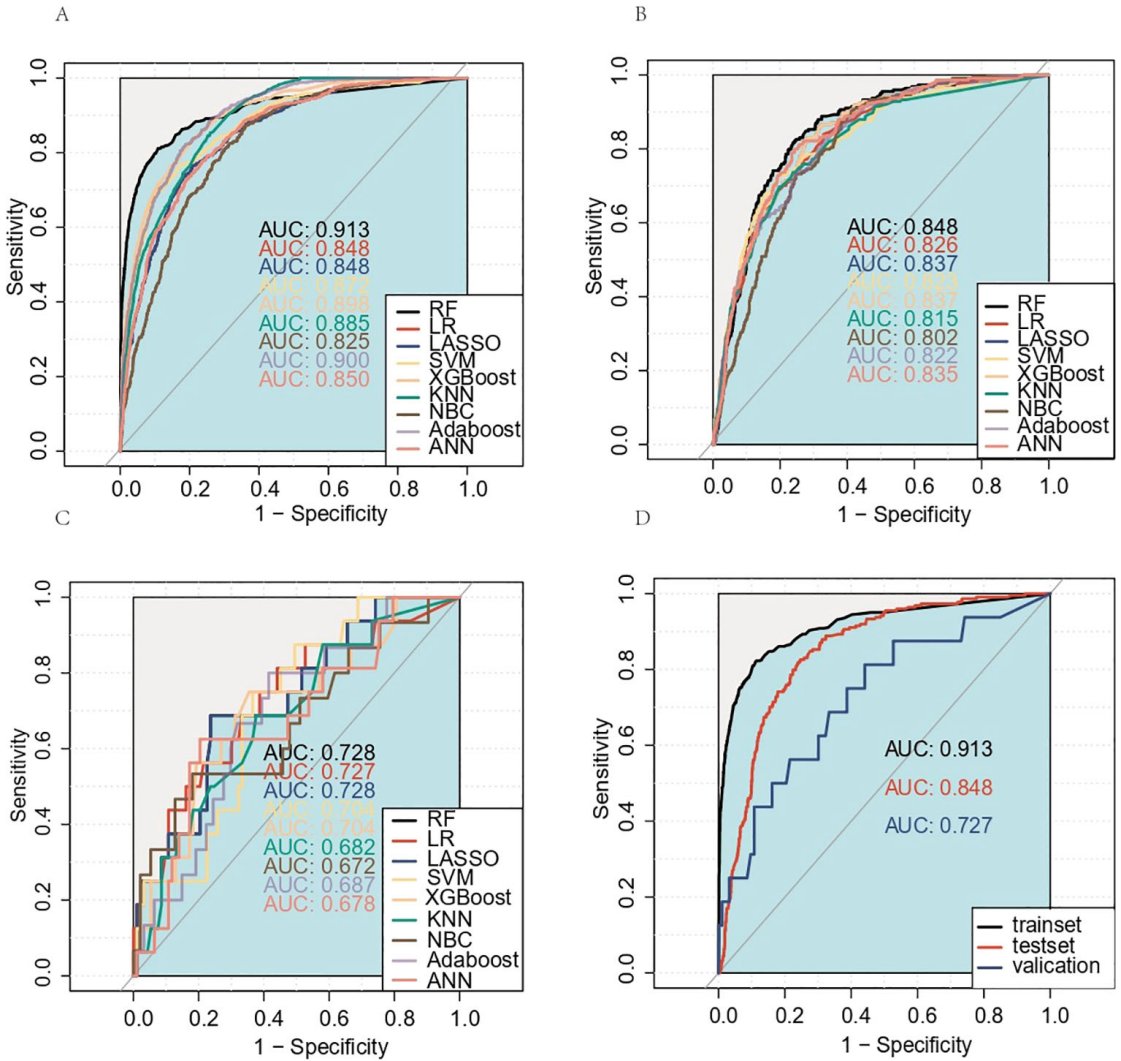
Receiver operating characteristic (ROC) curves for the training set, test set, and external validation set prediction models. **(A)** Training set; **(B)** test set; **(C)** external validation set. The aggregation of the previous ROC curves for the RF model **(D)**. AUC, area under the ROC curve; RF, random forest; LR, logistic regression; LASSO, least absolute shrinkage and selection operator; SVM, support vector machine; KNN, K-nearest neighbor; NBC, naive Bayes classifier; ANN, artificial neural network.

**Table 3** and **Figure 4A** present the evaluation standards for each model comparison, including ROC curves, specificity, sensitivity, accuracy, recall, and F1 score. Based on the comparison results, it is concluded that the random forest model has the highest predictive value. Its AUC (0.913), specificity (0.891), and accuracy (0.880) were the best among the nine models. The results in the testing set verified this point again. The AUC of the ROC curve for the RF model was 0.848 (**Figure 4B**), which was noticeably superior to those of the other eight models. Finally, the RF models were externally validated using our 109 hospital patients (AUC = 0.728) (**Figure 4C**). We also made an aggregation of the previous ROC curves (**Figure 4D**). In summary, we trained eight machine learning prediction models with data from the training set. Through the experimental results of the test set and validation set, it was determined that the RF model has a relatively accurate ability to predict the risk of DM in MDGA patients and has high clinical value.

**Table 3:** Comparison of the predictive performance values of nine forecasting models in the training set.

Models	AUC	Specificity	Sensitivity	Accuracy	Precision	Recall	F1 score
RF	0.913	0.891	0.811	0.880	0.548	0.811	0.654
LR	0.848	0.791	0.766	0.787	0.372	0.766	0.501
LASSO	0.848	0.791	0.766	0.787	0.372	0.766	0.501
SVM	0.872	0.834	0.760	0.823	0.425	0.760	0.545
XGBoost	0.989	0.792	0.836	0.798	0.394	0.836	0.536
KNN	0.885	0.740	0.853	0.756	0.348	0.853	0.494
NBC	0.825	0.641	0.870	0.673	0.282	0.870	0.426
AdaBoost	0.900	0.811	0.823	0.813	0.414	0.823	0.551
ANN	0.850	0.749	0.792	0.755	0.338	0.792	0.474

RF, random forest; LR, logistic regression; LASSO, least absolute shrinkage and selection operator; SVM, support vector machine; XGBoost, eXtreme Gradient Boosting; KNN, K-nearest neighbor; NBC, naive Bayesian model; AdaBoost, adaptive boosting; ANN, artificial neural network.

## 4 Prognostic analysis and prediction of MDGA patients with established DM

### 4.1 Patient baseline characteristics

The 749 eligible MDGA patients with DM were randomized into two groups in the same 7:3 split. The training set included 524 patients, while the testing set included 225 patients. In terms of clinical characteristics, 40–60 years of age was the most common age for distant metastasis according to the MDGA (55.41% according to the SEER data), and the highest proportion of distant metastases according to the MDGA originated in cardia (42.86% according to the SEER data). Descriptions of other clinical characteristics are summarized in the accompanying table (**Table 4**). The results suggested that no statistically significant differences were found between the basic information of the two datasets.

**Table 4:** Basic information about MDGA patients with DM.

Variable	Total (*n* = 749)	Train_set (*n* = 524)	Valid_set (*n* = 225)	Statistic	*P*
Survival months, *M* (Q_1_, Q_3_)	8.00 (3.00–18.00)	9.00 (3.00–19.00)	7.00 (2.00–16.00)	*Z* = 1.771	0.077
Age, *n* (%)				*χ*² = 4.450	0.217
<40	16 (2.14)	14 (2.67)	2 (0.89)		
40–60	169 (22.56)	123 (23.47)	46 (20.44)		
60–80	415 (55.41)	280 (53.44)	135 (60.00)		
>80	149 (19.89)	107 (20.42)	42 (18.67)		
Sex, *n* (%)				*χ*² = 0.022	0.882
Male	552 (73.7)	387 (73.85)	165 (73.33)		
Female	197 (26.3)	137 (26.15)	60 (26.67)		
T stage, *n* (%)				*χ*² = 0.592	0.898
1	214 (28.57)	152 (29.01)	62 (27.56)		
2	59 (7.88)	43 (8.21)	16 (7.11)		
3	257 (34.31)	179 (34.16)	78 (34.67)		
4	219 (29.24)	150 (28.63)	69 (30.67)		
N stage, *n* (%)				*χ*² = 3.476	0.324
0	249 (33.24)	179 (34.16)	70 (31.11)		
1	295 (39.39)	212 (40.46)	83 (36.89)		
2	96 (12.82)	62 (11.83)	34 (15.11)		
3	109 (14.55)	71 (13.55)	38 (16.89)		
Primary site, *n* (%)				*χ*² = 6.002	0.647
Body	69 (9.21)	46 (8.78)	23 (10.22)		
Cardia	321 (42.86)	221 (42.18)	100 (44.44)		
Fundus	31 (4.14)	25 (4.77)	6 (2.67)		
Gastric antrum	145 (19.36)	100 (19.08)	45 (20.00)		
Greater curvature	27 (3.6)	20 (3.82)	7 (3.11)		
Lesser curvature	51 (6.81)	34 (6.49)	17 (7.56)		
Overlapping lesion	46 (6.14)	35 (6.68)	11 (4.89)		
Pylorus	11 (1.47)	6 (1.15)	5 (2.22)		
Stomach	48 (6.41)	37 (7.06)	11 (4.89)		
Tumor size, *n* (%)				*χ*² = 2.719	0.437
<2	78 (10.41)	51 (9.73)	27 (12.00)		
2 to 5	374 (49.93)	271 (51.72)	103 (45.78)		
5 to 8	216 (28.84)	145 (27.67)	71 (31.56)		
>8	81 (10.81)	57 (10.88)	24 (10.67)		
Number of Reg LN, *n* (%)				*χ*² = 0.619	0.734
None	528 (70.49)	372 (70.99)	156 (69.33)		
1 to 3	27 (3.6)	20 (3.82)	7 (3.11)		
4 or more	194 (25.9)	132 (25.19)	62 (27.56)		
Chemotherapy, *n* (%)				*χ*² = 0.584	0.445
No/unknown	294 (39.25)	201 (38.36)	93 (41.33)		
Yes	455 (60.75)	323 (61.64)	132 (58.67)		
Radiation, *n* (%)				*χ*² = 0.496	0.481
None/unknown	575 (76.77)	406 (77.48)	169 (75.11)		
Beam radiation	174 (23.23)	118 (22.52)	56 (24.89)		
Cause, *n* (%)				*χ*² = 0.291	0.590
Alive or dead of other cause	141 (18.83)	96 (18.32)	45 (20.00)		
Dead (attributable to this cancer dx)	608 (81.17)	428 (81.68)	180 (80.00)		
Status, *n* (%)				*χ*² = 0.079	0.778
Alive	70 (9.35)	50 (9.54)	20 (8.89)		
Dead	679 (90.65)	474 (90.46)	205 (91.11)		
Survival months, *M* (Q_1_, Q_3_)	8.00 (3.00–18.00)	9.00 (3.00–19.00)	7.00 (2.00–16.00)	*Z* = 1.771	0.077

### 4.2 Analysis of prognosis-related factors

Using OS and CSS as prognostic endpoints, we performed univariate and multivariate Cox regression analyses on data from eligible patients screened from the SEER database. Nine variables were included in the univariate analysis, and the detailed results are shown in the left half of **Tables 5** and **6**. Afterward, according to the outcome, statistically significant variables were included in the multivariate analyses.

**Table 5:** Cox regression analysis of OS in the SEER cohort.

Variables	Beta	S.E	*Z*	*P*	HR (95% CI)	m_Beta	m_S.E	m_*Z*	a*P*	aHR (95% CI)
Age										
<40					Ref					Ref
40–60	0.19	0.29	0.66	0.506	1.21 (0.69–2.14)	0.13	0.29	0.45	0.655	1.14 (0.64–2.03)
60–80	0.36	0.28	1.27	0.204	1.43 (0.82–2.49)	0.18	0.29	0.63	0.526	1.20 (0.68–2.12)
>80	0.91	0.29	3.12	0.002	2.48 (1.40–4.38)	0.53	0.30	1.77	0.077	1.70 (0.94–3.07)
Sex										
Male					Ref					
Female	−0.00	0.09	−0.05	0.960	1.00 (0.84–1.18)					
T stage										
4					Ref					Ref
2	−0.20	0.15	−1.32	0.187	0.82 (0.61–1.10)	−0.33	0.16	−2.11	0.035	0.72 (0.53–0.98)
1	0.27	0.10	2.68	0.007	1.31 (1.08–1.60)	−0.16	0.11	−1.41	0.157	0.85 (0.69–1.06)
3	−0.19	0.10	−1.98	0.048	0.82 (0.68–0.99)	−0.29	0.10	−2.90	0.004	0.75 (0.61–0.91)
N stage										
3					Ref					
1	0.08	0.12	0.64	0.523	1.08 (0.85–1.37)					
0	0.21	0.12	1.70	0.089	1.23 (0.97–1.56)					
2	0.08	0.15	0.55	0.586	1.08 (0.81–1.45)					
Primary site										
Lesser curvature					Ref					Ref
Cardia	0.04	0.16	0.25	0.800	1.04 (0.76–1.42)	0.16	0.17	0.93	0.351	1.17 (0.84–1.62)
Fundus	0.65	0.24	2.78	0.005	1.92 (1.21–3.05)	0.50	0.24	2.10	0.036	1.65 (1.03–2.63)
Stomach	−0.14	0.22	−0.65	0.516	0.87 (0.56–1.33)	−0.44	0.22	−2.00	0.046	0.64 (0.41–0.99)
Gastric antrum	−0.00	0.17	−0.00	0.998	1.00 (0.71–1.40)	−0.04	0.17	−0.25	0.805	0.96 (0.68–1.35)
Overlapping lesion	0.06	0.21	0.26	0.797	1.06 (0.69–1.61)	−0.09	0.22	−0.40	0.691	0.92 (0.60–1.41)
Body	0.07	0.19	0.37	0.710	1.08 (0.73–1.57)	0.10	0.20	0.49	0.626	1.10 (0.75–1.63)
Greater curvature	−0.04	0.25	−0.15	0.879	0.96 (0.59–1.58)	−0.46	0.26	−1.78	0.075	0.63 (0.38–1.05)
Pylorus	−0.37	0.38	−0.97	0.334	0.69 (0.33–1.46)	−0.02	0.39	−0.05	0.959	0.98 (0.46–2.10)
Tumor size										
<2					Ref					
>8	0.02	0.17	0.10	0.923	1.02 (0.73–1.41)					
2 to 5	0.20	0.13	1.51	0.131	1.22 (0.94–1.57)					
5 to 8	0.11	0.14	0.80	0.426	1.12 (0.85–1.46)					
Number of Reg LN										
None					Ref					Ref
1 to 3	−0.24	0.21	−1.13	0.257	0.79 (0.52–1.19)	−0.60	0.22	−2.74	0.006	0.55 (0.35–0.84)
4 or more	−0.59	0.09	−6.49	<0.001	0.55 (0.46–0.66)	−0.78	0.10	−7.48	<0.001	0.46 (0.37–0.56)
Chemotherapy										
No/unknown					Ref					Ref
Yes	−1.05	0.08	−13.00	<0.001	0.35 (0.30–0.41)	−1.20	0.09	−12.76	<0.001	0.30 (0.25–0.36)
Radiation										
None/unknown					Ref					Ref
Beam radiation	−0.23	0.09	−2.53	0.011	0.79 (0.66–0.95)	−0.17	0.10	−1.82	0.068	0.84 (0.70–1.01)

**Table 6:** Cox regression analysis of CSS according to the SEER data.

Variables	Beta	S.E	*Z*	*P*	HR (95% CI)	m_Beta	m_S.E	m_*Z*	a*P*	aHR (95% CI)
Age										
<40					Ref					Ref
40–60	0.10	0.29	0.36	0.721	1.11 (0.63–1.96)	0.04	0.30	0.15	0.884	1.04 (0.58–1.86)
60–80	0.22	0.28	0.79	0.432	1.25 (0.72–2.18)	0.06	0.29	0.22	0.828	1.06 (0.60–1.88)
>80	0.75	0.29	2.55	0.011	2.11 (1.19–3.74)	0.39	0.30	1.28	0.201	1.48 (0.81–2.68)
Sex										
Male					Ref					
Female	0.00	0.09	0.04	0.965	1.00 (0.84–1.20)					
T stage										
4					Ref					Ref
2	−0.22	0.16	−1.37	0.172	0.80 (0.58–1.10)	−0.38	0.17	−2.28	0.023	0.68 (0.49–0.95)
1	0.29	0.11	2.70	0.007	1.33 (1.08–1.65)	−0.17	0.12	−1.44	0.150	0.84 (0.67–1.06)
3	−0.14	0.10	−1.39	0.166	0.87 (0.71–1.06)	−0.25	0.11	−2.29	0.022	0.78 (0.63–0.96)
N stage										
3					Ref					
1	0.08	0.13	0.65	0.519	1.09 (0.85–1.39)					
0	0.20	0.13	1.56	0.119	1.22 (0.95–1.58)					
2	0.13	0.15	0.83	0.407	1.14 (0.84–1.54)					
Primary site										
Lesser curvature					Ref					Ref
Cardia	0.02	0.16	0.11	0.916	1.02 (0.74–1.40)	0.11	0.17	0.65	0.516	1.12 (0.80–1.57)
Fundus	0.61	0.25	2.49	0.013	1.84 (1.14–2.99)	0.45	0.25	1.79	0.073	1.56 (0.96–2.55)
Stomach	−0.18	0.23	−0.79	0.428	0.83 (0.53–1.31)	−0.47	0.23	−2.01	0.045	0.63 (0.40–0.99)
Gastric antrum	−0.09	0.18	−0.50	0.616	0.91 (0.64–1.30)	−0.12	0.18	−0.67	0.500	0.88 (0.62–1.27)
Overlapping lesion	0.07	0.22	0.33	0.744	1.07 (0.70–1.66)	−0.08	0.22	−0.36	0.717	0.92 (0.59–1.43)
Body	0.02	0.20	0.09	0.932	1.02 (0.68–1.52)	0.02	0.21	0.12	0.908	1.02 (0.68–1.54)
Greater curvature	−0.38	0.29	−1.31	0.192	0.68 (0.38–1.21)	−0.81	0.30	−2.74	0.006	0.44 (0.25–0.79)
Pylorus	−0.30	0.39	−0.77	0.439	0.74 (0.35–1.58)	0.07	0.39	0.18	0.854	1.07 (0.50–2.32)
Tumor size										
<2					Ref					
>8	0.06	0.18	0.35	0.725	1.07 (0.75–1.51)					
2 to 5	0.26	0.14	1.86	0.062	1.30 (0.99–1.70)					
5 to 8	0.12	0.15	0.81	0.419	1.13 (0.84–1.51)					
Number of Reg LN										
None					Ref					Ref
1 to 3	−0.23	0.22	−1.07	0.287	0.79 (0.52–1.22)	−0.61	0.23	−2.65	0.008	0.54 (0.35–0.85)
4 or more	−0.68	0.10	−6.83	<0.001	0.51 (0.42–0.62)	−0.88	0.11	−7.82	<0.001	0.42 (0.33–0.52)
Chemotherapy										
No/unknown					Ref					Ref
Yes	−1.04	0.09	−12.28	<0.001	0.35 (0.30–0.42)	−1.24	0.10	−12.46	<0.001	0.29 (0.24–0.35)
Radiation										
None/unknown					Ref					Ref
Beam radiation	−0.22	0.10	−2.33	0.020	0.80 (0.66–0.97)	−0.16	0.10	−1.61	0.108	0.85 (0.70–1.04)

The Cox regression results for OS are shown in **Table 5**. The detailed outcomes suggested that age, T stage, primary site, chemotherapy, radiation, and the number of Reg LNs were correlated with OS in MDGA patients. Multifactorial analysis for OS revealed that only T stage (2 and 3), primary site, chemotherapy, and number of Reg LNs were statistically significant independent risk factors for MDGA patients with established DM. Moreover, patients with higher T stages (T3 and 4) and without chemotherapy had significantly shorter survival and worse outcomes. Patients with superficial primary sites (gastric antrum and greater curvature) and a greater number of Reg LNs could have improved outcomes. More comprehensive OS analysis information, such as the analytical CIs and *P*-values for each variable, is collated and displayed in **Table 5**.

The outcome of the Cox regression analysis using CSS as the endpoint is presented in **Table 6**. The primary site, number of Reg LNs, age, T stage, chemotherapy, and radiotherapy variables were integrated into the multifactorial analysis. The analysis indicated that the number of Reg LNs, T stage, primary site, and chemotherapy were considered statistically significant independent risk factors for CSS. The CIs and the corresponding *P*-values are summarized in **Table 6**.

### 4.3 Nomogram

**Figure 5:**
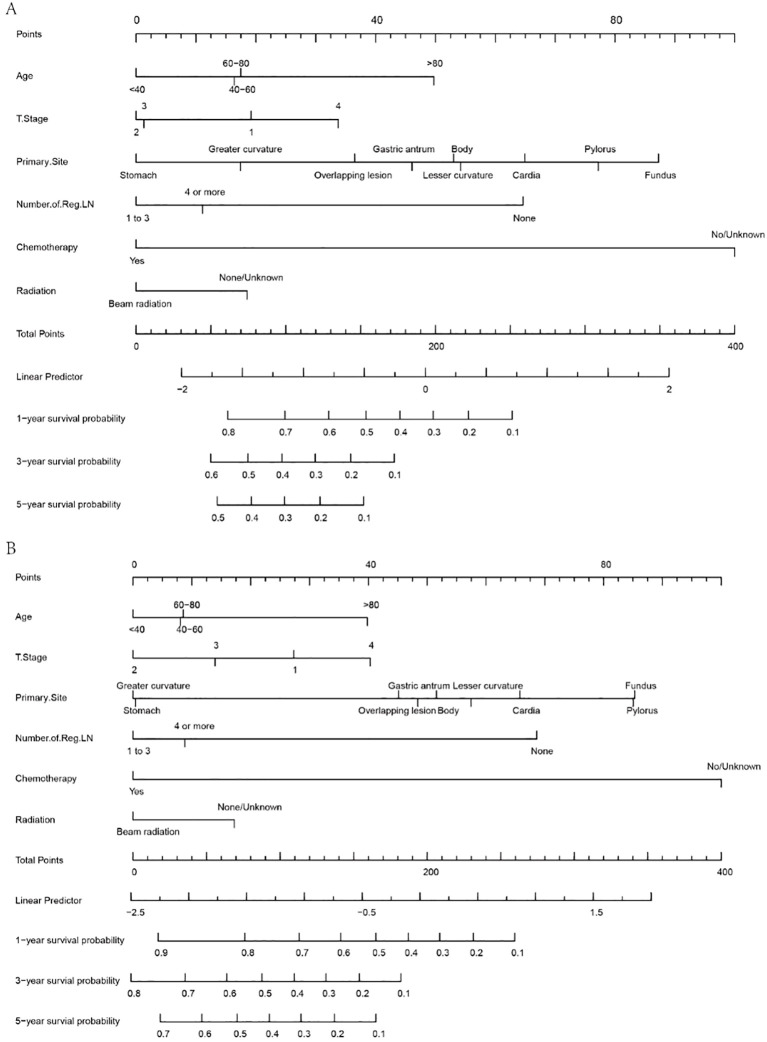
Nomograms for 1-, 3-, and 5-year OS **(A)** and CSS **(B)** in MDCA patients with DM.

According to the outcomes obtained in the previous steps, this study developed a visual nomogram to predict the survival time of MDGA patients with established DM. The nomogram, derived from the prognostically relevant risk factors that have been identified, provides a score based on the patient’s current condition. Physicians can assess a patient’s probability of 1-, 3-, and 5-year OS/CSS based on this nomogram (**Figure 5**). According to the OS nomogram (**Figure 5A**), of the five independent risk factors, chemotherapy had the greatest impact on survival, followed by the primary tumor site, while T stage had the least impact. According to the CSS nomogram (**Figure 5B**), the presence or absence of chemotherapy was considered to be the most influential factor for survival, followed by the lymph node positivity rate.

A simple example of how to use a nomogram is given below. Suppose a 60-year-old patient with distant metastases from MDGA has received conventional chemotherapy but no radiotherapy. At the same time, the pathological findings suggest that the tumor originated in the greater curvature, the current T stage is 3, and the number of regional LNs reaches more than four. At this point, an approximate score can be calculated based on the nomogram (age, 17.5 points; T stage 3, 2 points; primary site, 17 points; number of regional LNs, 11 points; received chemotherapy, 0; not received radiotherapy, 18 points). This hypothetical patient would therefore have a total score of 65.5, and this score was plotted against a scale of total scores. By plotting vertically on a straight line of survival probability, one can derive the probability that the overall survival available for reference is approximately 0.78, 0.55, and 0.45 for 1, 3, and 5 years, respectively. Similarly, the corresponding CSS for this patient can be calculated using the same steps as above.

### 4.4 Evaluation and validation of the nomograms

**Figure 6:**
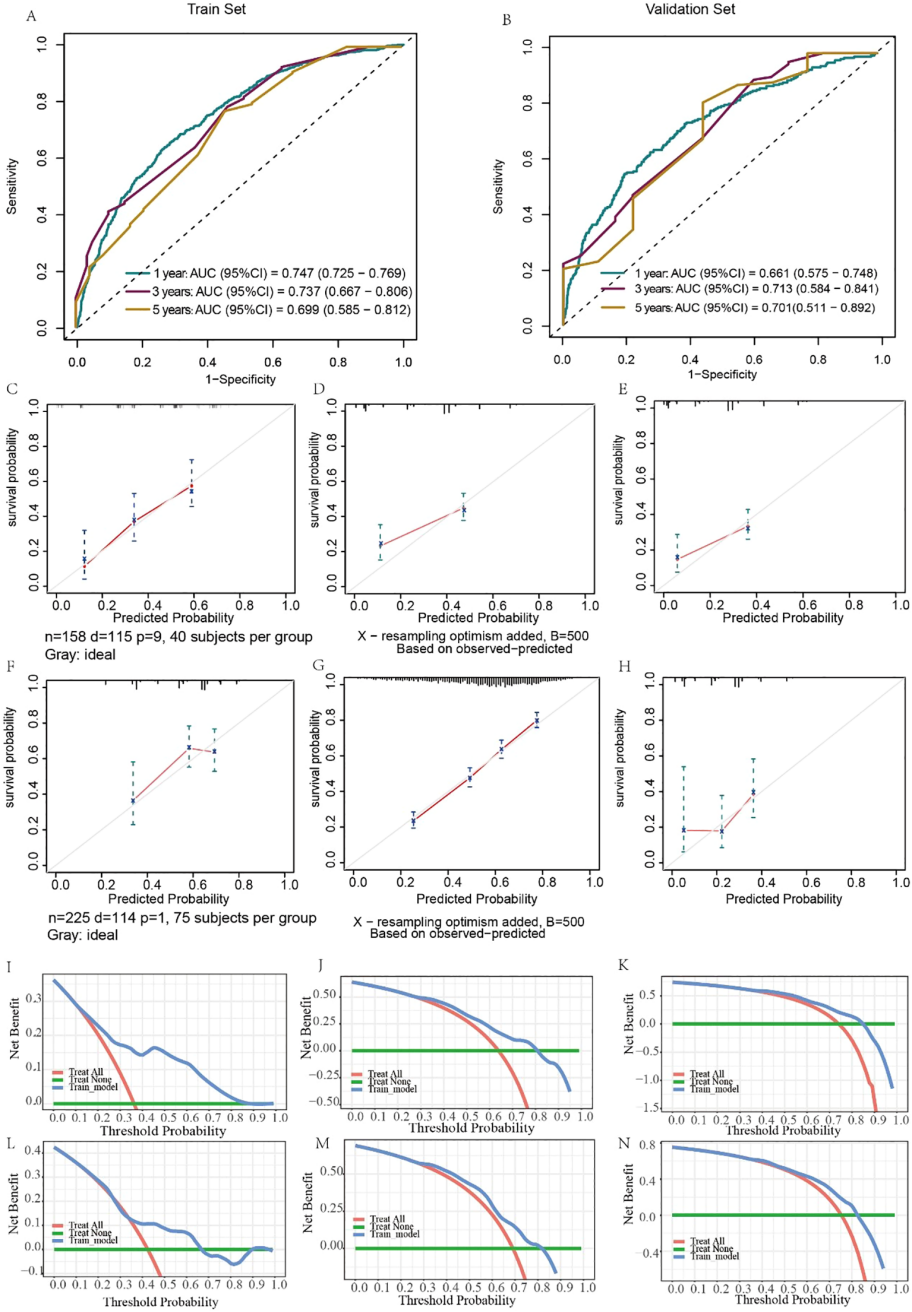
Evaluation of the ability of the nomogram to predict OS. ROC curves validating the OS prediction nomogram for 1-, 3-, and 5-year OS in the training set **(A)** and validation set **(B)**. Calibration curves validating the OS prediction nomograms for 1-, 3-, and 5-year OS in the training set **(C–E)** and validation set **(F–H)**. Decision curve analysis validating the OS prediction nomogram for 1-, 3-, and 5-year OS in the training set **(I–K)** and validation set **(L–N)**.

The predictive results and clinical value of the nomograms were assessed and verified by the C-index, AUC, calibration curve, and DCA. In the training set, the AUC values for predicting 1-, 3-, and 5-year OS were 0.797, 0.807, and 0.737, respectively (**Figure 6A**), while in the validation set, they were 0.757, 0.737, and 0.718, respectively (**Figure 6B**). The C-index of the nomograms was 0.726 (95% CI, 0.703–0.748) in the training set for OS and 0.703 (95% CI, 0.663–0.744) in the validation set. The fit of the 1-, 3-, and 5-year calibration curves for predicting OS was satisfactory (**Figures 6C–H**). In the calibration curves of the nomograms in the training and validation cohorts, the red curve fit line matches the gray diagonal line (representing the predicted probability of the ideal state) to a high degree, suggesting that the predicted probability of survival via the nomograms remains generally consistent with the observed probability of survival, with no excessive overestimation or underestimation of risk. The DCA curve presented graphically in **Figures 6I–N** suggests that this nomogram for OS has excellent net clinical efficacy.

**Figure 7:**
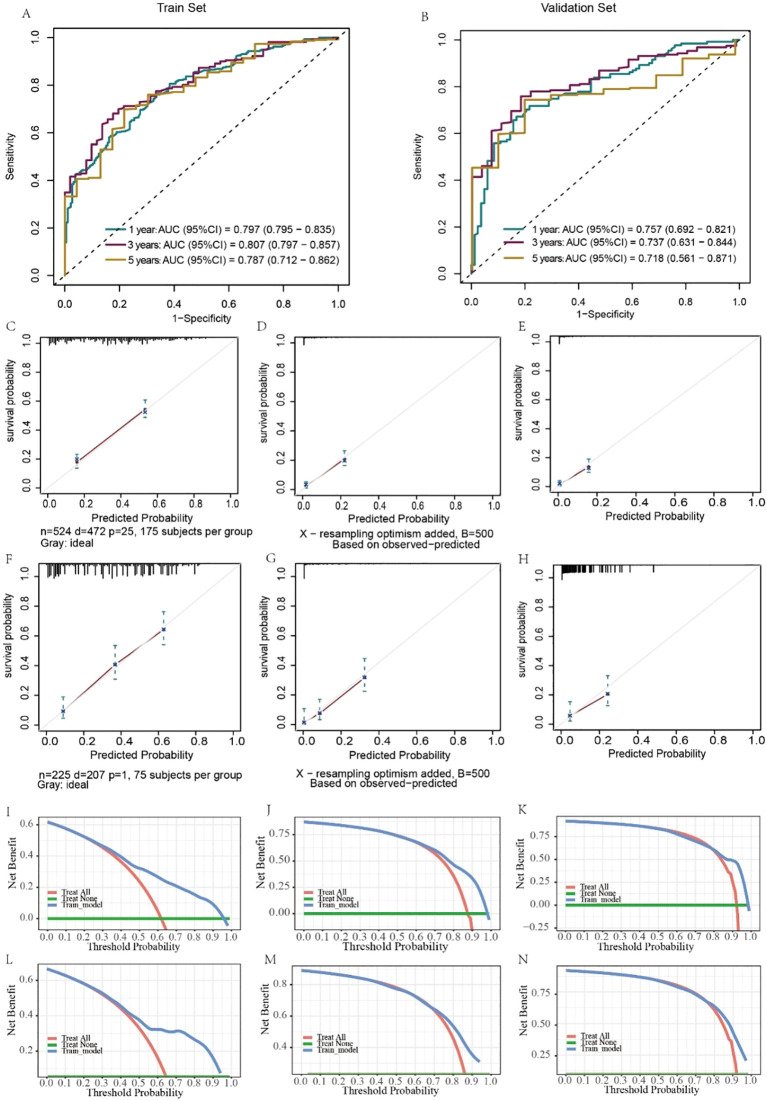
Evaluation of the ability of the nomogram to predict CSS. ROC curves validating the CSS prediction nomogram for 1-, 3-, and 5-year RFS in the training set **(A)** and validation set **(B)**. Calibration curves validating the CSS prediction nomograms for 1-, 3-, and 5-year survival in the training cohort **(C–E)** and validation cohort **(F–H)**. Decision curve analysis validating the CSS prediction nomogram for 1-, 3-, and 5-year RFS in the training set **(I–K)** and validation set **(L–N)**.

Similarly, the results for evaluating the CSS nomograms show a positive applicability. The C-index was 0.727 (95% CI, 0.703–0.751) for the training set and 0.705 (95% CI, 0.663–0.748) for the validation set. In addition, the AUCs of the nomograms were 0.747, 0.737, and 0.699 for 1-, 3-, and 5-year CSS, respectively, in the training cohort (**Figure 7A**), and in the validation cohort, the AUCs were 0.661, 0.713, and 0.892, respectively (**Figure 7B**). Moreover, both the calibration curves and DCA curves used for the 1-, 3-, and 5-year CSS forecasts also exhibited satisfactory fits and net gains (**Figures 7C–N**). In summary, the nomograms produced to predict the prognosis of MDGA patients with DM had considerable discriminatory and calibrating power.

## 5 Analysis of the impact of more detailed LN indicators on the occurrence of DM and prognosis of MDGA

The above studies have suggested a strong association between multiple lymph node indices and DM and the prognosis of MDGA. Although good predictive efficacy can be achieved by categorizing the number of positive LNs (0, 1 to 3, 3+), 70% of patients in the database had a positive lymph node clearance of 0. This suggests that the existing lymph node indices may not describe a patient’s prognosis specifically; thus, more diversified ways of evaluating the metastasis and immune mechanisms of patients are needed. Lymph node positivity, the specific number of negative/positive lymph nodes, and visualization of LN metastasis may be better indicators of DM risk and survival; therefore, we collected more detailed data from our institution and performed a logistic analysis to identify risk factors associated with DM.

We collected data from 109 patients with moderately differentiated gastric adenocarcinoma in our hospital. Data such as LODDS and the number of Neg LNs were analyzed and calculated, followed by logistic regression to explore the risk factors for distant metastasis in patients with MDGA and Cox regression to analyze the risk factors affecting the prognosis of patients with MDGA. Our univariate logistic regression results showed that the number of negative LNs and the LODDS were considered to be influential factors for the occurrence of DM in MDGA (**Table 7**). However, it is noteworthy that in regard to our multifactor logistic regression analysis of the variables of interest, our results lacked statistical significance. We conducted single- and multivariate Cox analyses of our patient data. As shown in **Table 8**, 15 variables were included. The results of the univariate analysis revealed that nine variables, including the number of Reg LNs, LNR, age >80 years, TNM stage, tumor size, gross LN metastasis, and number of Reg LNs, had an impact on the prognosis of MDGA patients (*P* < 0.05). These findings were subsequently incorporated into a multifactorial analysis, which indicated that the LNR, T stage (1 and 2), and gross LN metastasis 3 cm away from the tumor were independent risk factors, whereas the number of Reg LNs and the number of Reg LNs in groups 1–3 were considered protective factors. More specific data are shown in **Tables 7** and **8**.

**Table 7:** The risk factors for developing DM in MDGA patients were analyzed by logistic regression based on our data.

Variables	Beta	S.E	*Z*	OR (95% CI)	*P*	aBeta	aS.E	a*Z*	aOR (95% CI)	a*P*
Number of all LNs	−0.03	0.02	−1.27	0.97 (0.93–1.02)	0.203					
Number of Reg LN	0.05	0.04	1.50	1.06 (0.98–1.13)	0.135					
Number of Neg LN	−0.06	0.03	−2.00	0.94 (0.89–0.99)	0.046	−0.04	0.03	−1.48	0.96 (0.90–1.01)	0.138
LODDS	−3.08	1.48	−2.09	0.05 (0.00–0.83)	0.037	−1.68	1.59	−1.05	0.19 (0.01–4.24)	0.292
LNR	0.94	1.18	0.80	2.57 (0.25–25.89)	0.423					
Number of Reg LN_group										
None				1.00 (reference)						
1 to 3	−0.10	0.74	−0.14	0.90 (0.21–3.88)	0.890					
4 or more	−0.20	0.63	−0.32	0.82 (0.24–2.80)	0.747					
Gross LN metastasis										
None				1.00 (reference)						
3 cm away from the tumor	0.69	0.68	1.02	2.00 (0.52–7.62)	0.310					
Within 3 cm of the tumor	−1.11	0.81	−1.38	0.33 (0.07–1.60)	0.169					
Age										
<40				1.00 (reference)						
40–60	−0.95	1.27	−0.75	0.39 (0.03–4.67)	0.455					
60–80	−0.76	1.21	−0.63	0.47 (0.04–4.98)	0.527					
>80	0.41	1.68	0.24	1.50 (0.06–40.63)	0.810					
Sex										
Male				1.00 (Reference)						
Female	−0.26	0.69	−0.38	0.77 (0.20–2.98)	0.707					
T stage										
1				1.00 (reference)					1.00 (reference)	
2	0.27	1.45	0.19	1.31 (0.08–22.62)	0.851	0.24	1.48	0.16	1.27 (0.07–23.15)	0.872
3	1.25	1.09	1.15	3.50 (0.41–29.78)	0.251	1.27	1.12	1.13	3.56 (0.39–32.31)	0.259
4	2.46	1.17	2.11	11.67 (1.19–114.57)	0.035	2.34	1.21	1.93	10.40 (0.97–111.50)	0.053
N stage										
0				1.00 (reference)						
1	1.06	0.73	1.45	2.88 (0.69–12.00)	0.146					
2	1.54	0.90	1.72	4.67 (0.81–26.98)	0.085					
3	1.13	0.98	1.15	3.11 (0.45–21.40)	0.249					
Primary site										
Body				1.00 (reference)						
Cardia	16.83	2,465.33	0.01	20,408,610.53 (0.00–Inf)	0.995					
Gastric antrum	15.93	2,465.33	0.01	8,260,628.07 (0.00–Inf)	0.995					
Lesser curvature	18.06	2,465.33	0.01	69,389,275.80 (0.00–Inf)	0.994					
Greater curvature	18.57	2,465.33	0.01	115,648,792.99 (0.00–Inf)	0.994					
Overlapping lesion	16.17	2,465.33	0.01	10,513,526.64 (0.00–Inf)	0.995					
Stomach	17.47	2,465.33	0.01	38,549,597.66 (0.00–Inf)	0.994					
Pylorus	0.00	4,088.28	0.00	1.00 (0.00–Inf)	1.000					
Fundus	18.57	2,465.33	0.01	115,648,792.99 (0.00–Inf)	0.994					
Tumor size										
<2				1.00 (reference)						
2 to 5	−0.04	0.90	−0.05	0.96 (0.16–5.63)	0.961					
5 to 8	1.06	0.89	1.19	2.89 (0.50–16.67)	0.234					
>8	1.84	0.97	1.90	6.29 (0.94–41.96)	0.058					
Chemotherapy										
No/unknown				1.00 (reference)						
Yes	0.39	0.56	0.69	1.47 (0.49–4.42)	0.488					

LODDS, log odds of metastatic lymph nodes; LNR, lymph node ratio.

**Table 8:** Cox regression analysis of risk factors affecting patient OS based on our data.

Variables	Beta	S.E	*Z*	*P*	HR (95% CI)	m_Beta	m_S.E	m_*Z*	a*P*	aHR (95% CI)
Number of all LNs	−0.02	0.01	−1.35	0.179	0.98 (0.96–1.01)					
Number of Reg LN	0.05	0.02	2.95	0.003	1.05 (1.02–1.08)	−0.12	0.05	−2.68	0.007	0.89 (0.81–0.97)
Number of Neg LN	−0.06	1.02	−0.06	0.950	0.94 (0.13–6.91)					
LODDS	0.25	0.33	0.78	0.436	1.29 (0.68–2.44)					
LNR	1.94	0.52	3.76	<0.001	6.93 (2.53–19.01)	5.09	1.67	3.04	0.002	162.60 (6.12–4,318.06)
Age										
<40					Ref					Ref
40–60	0.24	1.04	0.23	0.816	1.28 (0.16–9.88)	−1.36	1.27	−1.07	0.285	0.26 (0.02–3.10)
60–80	0.64	1.02	0.63	0.528	1.90 (0.26–13.92)	−0.48	1.23	−0.39	0.698	0.62 (0.06–6.93)
>80	2.46	1.16	2.12	0.034	11.71 (1.20–114.06)	1.59	1.47	1.08	0.279	4.92 (0.28–88.06)
Sex										
Male					Ref					
Female	−0.04	0.35	−0.11	0.910	0.96 (0.49–1.89)					
T stage										
1					Ref					Ref
2	−1.49	1.10	−1.36	0.173	0.22 (0.03–1.92)	−2.21	1.17	−1.90	0.058	0.11 (0.01–1.07)
3	1.15	0.48	2.38	0.017	3.15 (1.22–8.13)	−0.08	0.60	−0.14	0.890	0.92 (0.28–3.00)
4	1.36	0.56	2.43	0.015	3.89 (1.30–11.66)	−0.41	0.66	−0.63	0.532	0.66 (0.18–2.42)
N stage										
0					Ref					Ref
1	1.23	0.38	3.20	0.001	3.42 (1.61–7.26)	1.72	0.74	2.31	0.021	5.56 (1.30–23.90)
2	2.11	0.48	4.41	<0.001	8.25 (3.23–21.08)	2.13	0.88	2.42	0.016	8.42 (1.50–47.32)
3	1.00	0.55	1.82	0.069	2.72 (0.93–7.99)	1.37	1.03	1.33	0.183	3.94 (0.52–29.61)
M stage										
0					Ref					Ref
1	1.04	0.35	2.99	0.003	2.82 (1.43–5.58)	0.79	0.50	1.56	0.120	2.19 (0.82–5.90)
Primary site										
Body					Ref					
Cardia	0.16	0.62	0.27	0.791	1.18 (0.35–3.97)					
Gastric antrum	−0.16	0.65	−0.24	0.809	0.86 (0.24–3.03)					
Lesser curvature	0.12	0.76	0.16	0.875	1.13 (0.25–5.05)					
Greater curvature	1.33	0.92	1.45	0.148	3.78 (0.62–22.84)					
Overlapping lesion	−1.02	0.91	−1.11	0.266	0.36 (0.06–2.17)					
Stomach	0.11	0.91	0.12	0.901	1.12 (0.19–6.72)					
Pylorus	−0.64	1.16	−0.55	0.582	0.53 (0.05–5.10)					
Fundus	−16.30	3,293.13	−0.00	0.996	0.00 (0.00–Inf)					
Tumor size										
<2					Ref					Ref
2 to 5	1.83	0.74	2.48	0.013	6.25 (1.47–26.61)	1.60	0.92	1.75	0.081	4.94 (0.82–29.73)
5 to 8	2.33	0.76	3.08	0.002	10.30 (2.34–45.40)	1.43	0.91	1.57	0.116	4.19 (0.70–24.97)
>8	2.38	0.79	3.01	0.003	10.78 (2.29–50.84)	1.62	0.92	1.77	0.077	5.08 (0.84–30.76)
Chemotherapy										
No/unknown					Ref					
Yes	−0.10	0.31	−0.32	0.747	0.91 (0.50–1.65)					
Gross LN metastasis										
None					Ref					Ref
3 cm away from the tumor	1.40	0.47	2.99	0.003	4.04 (1.62–10.09)	1.96	0.66	2.97	0.003	7.11 (1.95–25.99)
Within 3 cm of the tumor	0.72	0.47	1.54	0.124	2.05 (0.82–5.13)	1.21	0.64	1.88	0.060	3.36 (0.95–11.90)
Number of Reg LN group										
None					Ref					Ref
1 to 3	0.98	0.40	2.43	0.015	2.66 (1.21–5.87)	−1.67	0.80	−2.08	0.038	0.19 (0.04–0.91)
4 or more	1.06	0.36	2.99	0.003	2.90 (1.44–5.82)	−1.45	0.88	−1.65	0.098	0.23 (0.04–1.31)

## 6 Discussion

Moderately differentiated gastric adenocarcinoma is common in clinical practice and has a high risk of metastasis and individual variability (34). Once a patient develops DM, the prognosis becomes extremely poor (35, 36). The OS of MDGA patients without DM is generally considered to be more than 22.3 months after surgical treatment (37). However, after the onset of DM, survival decreases in patients receiving conventional chemotherapy, with a median overall survival of just under 12 months (22, 38). Determining whether a patient has distant metastases is therefore particularly important and is vital for providing individualized prevention and treatment strategies in the clinic. In addition, the current prognostic method for patients with DM is relatively limited, and some DM-related indices, especially lymph node indices such as the LNR and LODDS, are considered to be important indicators of prognosis (39, 40). However, its specific clinical effects have still not been extensively and comprehensively tested.

Our major objectives for the investigation were to develop a forecasting system to predict the development of DM in persons with MDGA and to analyze the risk factors influencing the prognosis of persons with DM. In addition, this study analyzed the specific ability of six lymph node indicators in our patients to predict DM and prognosis using logistic and Cox regression. Nine machine learning samples were utilized for predicting distant metastases, with the RF model considered the most effective. Multivariate Cox regression analysis for MDGA patients who already had DM indicated that higher T stage (2 and 3), primary site, chemotherapy, and number of Reg LNs were independent risk factors for prognosis. Moreover, specialized nomograms created from our analysis results were evaluated and tested to show convincing prognostic discrimination and calibration capabilities.

Notably, the categorization of the number of positive LNs (0, 1 to 3, 4 or more) in the SSER database may achieve good predictive efficacy. However, nearly 70% of the patients in the database had a positive lymph node clearance of 0. This suggests that our single reliance on lymph node clearance results may not be effective in characterizing the prognosis of patients, and more diverse classifications and metrics are needed to evaluate a patient’s metastatic and prognostic condition. In this study, based on our patient data, we revealed that the LNR, gross LN metastasis, and the number of Reg LNs were found to be independent factors influencing the prognosis of MDGA patients with DM.

It is worth noting that the AUC of the validation set in this paper is generally slightly lower than that of the training set, which is a relatively common phenomenon, and the possible reasons are that the training set adopts the U.S. population samples from the SEER database, and extrapolation is not strong enough in the Chinese population, or the sample size of the validation set is not sufficient. In future research, we will further consider the extrapolation of the population and the adequacy of the samples to deepen and improve the prediction ability of the validation set.

Nevertheless, it must be noted that the research has been limited by its retrospective nature. Although the SEER database is very detailed and reliable, there are some more exhaustive data that it is unable to provide (41). For example, data on some noteworthy laboratory tests were not included, and some of the more nuanced pictures of the lymph nodes, as previously mentioned, were lacking. Furthermore, for the practical application of the nomogram, additional clinical information must be considered, including the ethnicity of the patient, their geographical location, and other pertinent factors. These data, which are absent from the database and not included in the study, have an impact on the results, and more information is required to enhance the nomogram. For our data, because of the sample size and other reasons, it is not as effective as it should be in carrying out some statistical studies, and in the future, it is necessary to collect more case and patient information for more in-depth analysis and studies.

## 7 Conclusion

In conclusion, this research investigated the variables linked to the development of DM in MDGA, including T stage, N stage, primary site, tumor size, number of positive LNs, and chemotherapy. Then, we investigated the prognostic factors, including T stage, primary location, chemotherapy, and number of Reg LNs, in MDGA patients with DM. Additionally, based on the prognostic analysis, separate nomograms of OS and CSS were produced for relevant influencing factors. Finally, the effect of multiple lymph node indicators on the metastasis and prognosis of MDGA patients was investigated. This study provides a reference for subsequent clinical studies and further suggests the importance of lymph node indicators.

## Data Availability

The raw data supporting the conclusions of this article will be made available by the authors, without undue reservation.
