# Correction: Machine learning to predict distant metastasis and prognostic analysis of moderately differentiated gastric adenocarcinoma patients: a novel focus on lymph node indicators

**DOI:** 10.3389/fimmu.2026.1932683

**Published:** 2026-07-15

**Authors:** Frontiers Production Office

**Affiliations:** Frontiers Media SA, Lausanne, Switzerland

**Keywords:** autonomic nervous system, bradycardia, diving reflex, heart rate, SCUBA

An incorrect **Funding** statement was provided. The correct funder is the “Science and Technology Program (No. 2024B0057) of the Jiangxi Provincial Administration of Traditional Chinese Medicine/” the correct Funding statement reads:

“This work was supported by the Science and Technology Program (No. 2024B0057) of the Jiangxi Provincial Administration of Traditional Chinese Medicine.”

The original version of this article has been updated.

