# Supplementary material for: Machine learning to predict distant metastasis and prognostic analysis of moderately differentiated gastric adenocarcinoma patients: a novel focus on lymph node indicators
Source: Front Immunol. 2024 Sep 19;15:1398685. doi: 10.3389/fimmu.2024.1398685 (PMC11446832; doi:10.3389/fimmu.2024.1398685)
Supplement: Supplementary file 1 [file Table1.docx]

Supplementary Material

# Supplementary Tables

**Supplementary Table 1 Results of forward regression analysis**

| **Variable** | **Estimate** | **Std. Error** | **z value** | **Pr(>\|z\|)** |
| --- | --- | --- | --- | --- |
| (Intercept) | -1.50638 | 0.46289 | -3.254 | 0.001137 |
| as.factor (Age) 40-60 | -0.417 | 0.44381 | -0.94 | 0.347428 |
| as.factor (Age) 60-80 | -0.77756 | 0.4356 | -1.785 | 0.074257 |
| as.factor (Age) >80 | -1.18581 | 0.45084 | -2.63 | 0.008533 |
| as.factor (Sex) Female | -0.11095 | 0.12479 | -0.889 | 0.373972 |
| as.factor (T.Stage) 2 | -0.64439 | 0.21298 | -3.026 | 0.002481 |
| as.factor (T.Stage) 3 | -0.19435 | 0.15581 | -1.247 | 0.212278 |
| as.factor (T.Stage) 4 | 0.69956 | 0.17334 | 4.036 | 5.44E-05 |
| as.factor (N.Stage) 1 | 1.00547 | 0.14134 | 7.114 | 1.13E-12 |
| as.factor (N.Stage) 2 | 0.87642 | 0.19882 | 4.408 | 1.04E-05 |
| as.factor (N.Stage) 3 | 1.80605 | 0.21145 | 8.541 | < 2e-16 |
| as.factor (Primary.Site) Gastric antrum | 0.68427 | 0.16909 | 4.047 | 5.19E-05 |
| as.factor (Primary.Site) Lesser curvature | 0.2554 | 0.21977 | 1.162 | 0.24519 |
| as.factor (Primary.Site) Pylorus | -0.05686 | 0.394 | -0.144 | 0.885256 |
| as.factor (Primary.Site) Body | 0.67253 | 0.20497 | 3.281 | 0.001034 |
| as.factor (Primary.Site) Greater curvature | 0.45201 | 0.29457 | 1.534 | 0.124909 |
| as.factor (Primary.Site) Stomach | 0.87829 | 0.23745 | 3.699 | 0.000217 |
| as.factor (Primary.Site) Overlapping lesion | 0.12073 | 0.26522 | 0.455 | 0.648945 |
| as.factor (Primary.Site) Fundus | 0.13823 | 0.27793 | 0.497 | 0.61894 |
| as.factor (Tumor.Size) 2 to 5 | 0.90691 | 0.17527 | 5.174 | 2.29E-07 |
| as.factor (Tumor.Size) 5 to 8 | 1.28066 | 0.19633 | 6.523 | 6.89E-11 |
| as.factor (Tumor.Size) >8 | 1.18192 | 0.24336 | 4.857 | 1.19E-06 |
| as.factor (Number.of.Reg.LN) 1 to 3 | -1.4454 | 0.26686 | -5.416 | 6.08E-08 |
| as.factor (Number.of.Reg.LN) 4 or more | -2.84351 | 0.14497 | -19.614 | < 2e-16 |
| as.factor (Chemotherapy) Yes | 0.19192 | 0.12871 | 1.491 | 0.­­­135951 |
